# Surgical treatment of primary hepatic neuroendocrine tumor diagnosed by Al^18^F-NOTA-Octreotide PET/CT: a case report

**DOI:** 10.3389/fmed.2023.1256176

**Published:** 2023-11-23

**Authors:** Zhongyan Zhang, Hehe Li

**Affiliations:** ^1^Department of Hepatobiliary Surgery, Weifang People's Hospital, Weifang, China; ^2^Department of Geriatrics, Weifang People's Hospital, Weifang, China

**Keywords:** hepatic primary neuroendocrine tumor, PHNETs, [^18^F]AlF-OC, PET/CT, diagnosis, surgical resection, case report

## Abstract

Neuroendocrine tumors (NETs) are a heterogeneous group of tumors originating from peptide-producing neurons and neuroendocrine cells. The liver is the most common site of metastasis for NETs, while primary hepatic neuroendocrine tumors (PHNETs) are exceedingly rare. While somatostatin receptor scintigraphy (SRS) has demonstrated superior efficacy compared to [^18^F]FDG PET imaging in the diagnosis of neuroendocrine tumors, [^18^F]AlF-NOTA-Octreotide ([^18^F]AlF-OC) PET/CT also exhibits specific advantages over SRS. This article presents a case study of a patient with a liver mass who underwent sequential [^18^F]FDG and [^18^F]AlF-OC PET/CT scans, ruling out hepatocellular carcinoma and confirming the diagnosis of PHNETs. Subsequently, the patient underwent surgical treatment. From another perspective, [^18^F]AlF-OC exhibits distinct advantages. The postoperative pathology revealed a PHNETs, which further emphasizes its clinical rarity.

## Introduction

Neuroendocrine tumors (NETs) are a heterogeneous group of tumors originating from peptide-producing neurons and neuroendocrine cells. They predominantly occur in the gastrointestinal tract, pancreas, and bronchopulmonary tissues. The liver is the most common site of metastasis for NETs, while primary hepatic neuroendocrine tumors (PHNETs) are exceedingly rare (1), accounting for only 0.8% of all NETs. The incidence of PHNETs is very low, accounting for only 0.46% of all primary liver tumors. These tumors typically occur in adults over 40, with a slightly higher prevalence in females than males.

PHNETs can occur as solitary or multiple lesions, and they typically present as large lesions with heterogeneous cystic and solid components. Common internal features include cystic changes and areas of necrosis, which contribute to the uneven density or signal characteristics observed. PET (Positron Emission Tomography) has the advantage of providing comprehensive information about the systemic tumor burden and the metabolic activity of the tumor. However, its diagnostic value for cystic lesions is limited. Numerous studies have demonstrated that Ga68 SSA-PET([^68^Ga]Ga-DOTANOC, [^68^Ga]Ga-DOTATOC, [^68^Ga]Ga-DOTATATE) imaging offers higher diagnostic accuracy for NETs (2–5). While 68Ga labeling has the advantage of eluting isotopes from a generator, fluorine labeling offers benefits like longer half-life, improved spatial resolution, higher production yield, and consistent advantages in imaging and logistics. [^18^F]AlF-OC demonstrates its advantages in certain aspects, particularly in diagnosing PHNETs.

Hepatic lobectomy is considered the most effective treatment for PHNETs. In some cases, a complete cure can be achieved through surgical intervention, leading to a favorable prognosis.

This article presents a case study of a patient with a liver mass who underwent sequential [^18^F]FDG and [^18^F]AlF-OC PET/CT scans, ruling out hepatocellular carcinoma and confirming the diagnosis of PHNETs. Subsequently, the patient underwent surgical treatment. In addition, a literature review was conducted to provide insights and guidance regarding treatment strategies for patients with PHNETs.

## Case description

**Figure 1:**
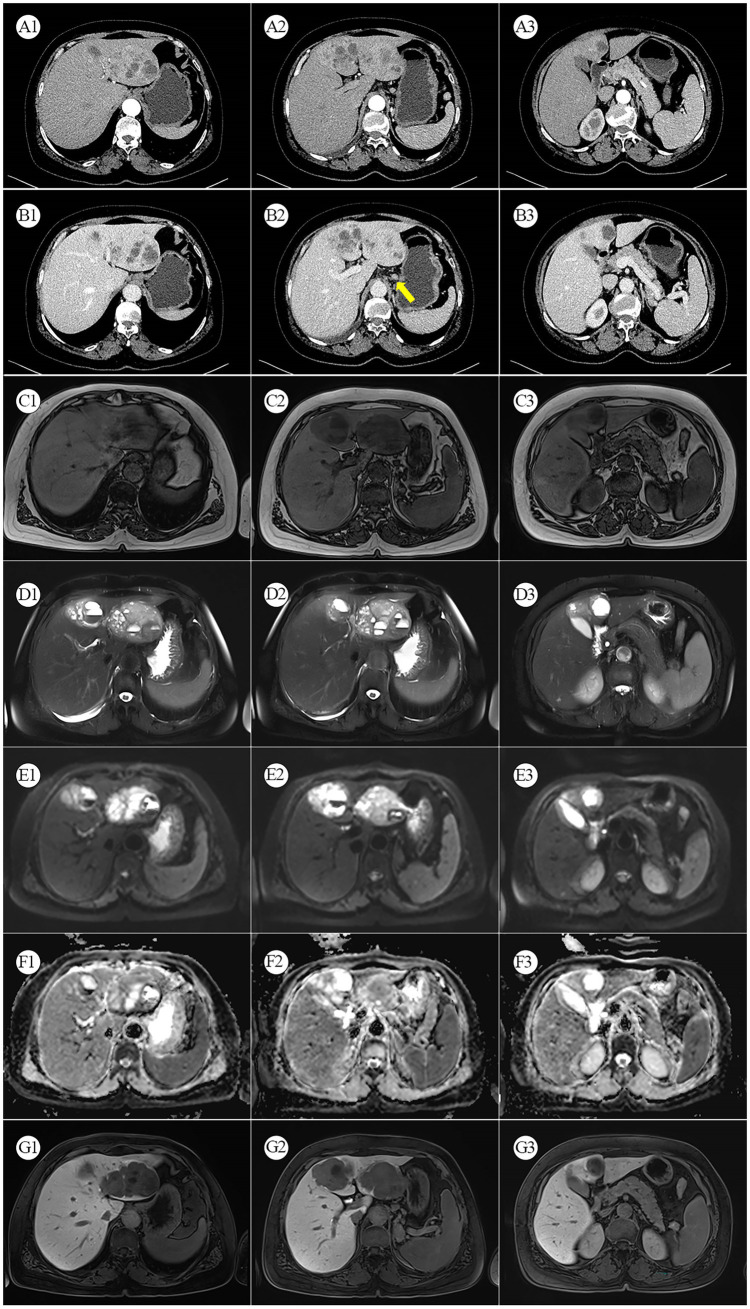
**(A1–A3)** Arterial phase CT. **(B1–B3)** Venous phase CT: The yellow arrows indicate enhanced and enlarged lymph nodes between the liver and stomach. **(C1–C3)** MRI T1-weighted image. **(D1–D3)** MRI T2-weighted image. **(E1–E3)** Diffusion-weighted MRI. **(F1–F3)** MRI ADC map. **(G1–G3)** Gadoxetic Acid-enhanced (Primovist) MRI.

**Figure 2:**
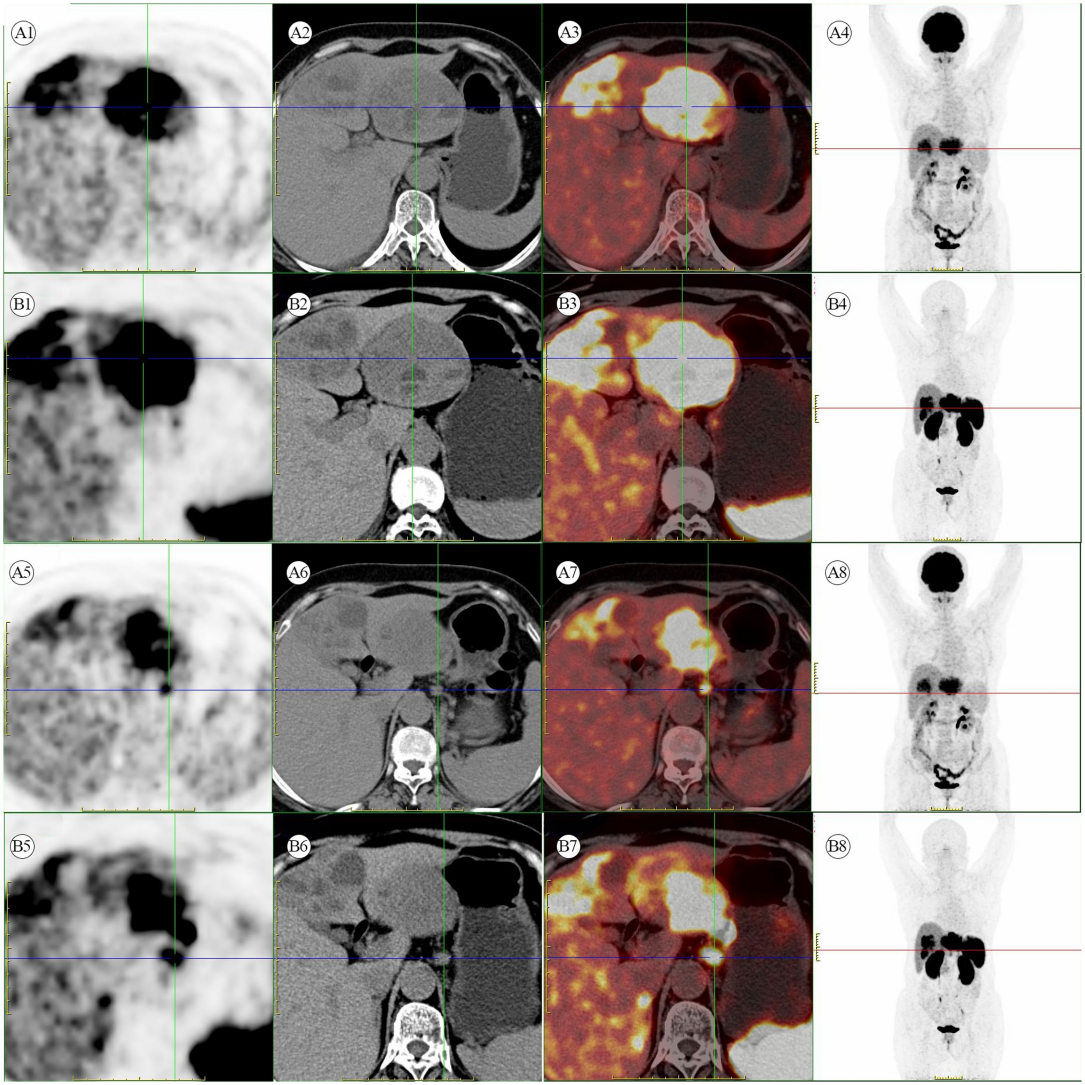
**(A1–A8)** [^18^F]FDG PET/CT scan. Multiple hypermetabolic heterogeneous lesions are present in the left lobe of the liver, indicating possible malignant lesions. Additionally, multiple hypermetabolic enlarged lymph nodes are seen in the space between the liver and stomach, suggesting metastasis. **(B1–B8)** [^18^F]AlF-OC PET/CT scan. A liver mass with multiple enlarged lymph nodes in the hepatogastric ligament shows high somatostatin receptor expression.

A 56-year-old female patient was found to have a liver mass during a physical examination on August 3, 2022. There is no previous history of chronic liver disease. Six years ago, she underwent a hysterectomy for uterine fibroids at a local hospital. On admission, no significant positive physical signs were detected during the examination. The results of the blood tests are as follows: Complete Blood Count (CBC), liver function, kidney function, electrolytes, and coagulation function are generally within normal ranges. The C-reactive protein (CRP) level is elevated at 25.7 mg/L. The hepatitis B panel shows positive results for anti-HBc, while the remaining parameters are negative. The testing for hepatitis C antibodies does not show any abnormalities. HIV and syphilis testing results are negative. On August 13, 2022, the patient underwent a contrast-enhanced abdomen CT, which revealed that the left lobe of the liver shows a clustered low-density mass with relatively clear borders. Within the mass, there are multiple rounded cystic areas with unclear boundaries. On the contrast-enhanced scan, the lesion demonstrates continuous enhancement. Additionally, the abdominal cavity has enlarged lymph nodes (Figure 1). A metastatic malignant tumor cannot be ruled out. On August 16, 2022, the patient underwent an [^18^F]FDG PET/CT scan, revealing a malignant tumor in the liver. Multiple clustered low-density lesions with heterogeneous enhancement are seen in the left lobe of the liver. The radioactive distribution is uneven with the maximum standardized uptake value (SUVmax) of 15.1, the mean standardized uptake value (SUVmean) of 9.3, and the tumor-to-normal (T/N) ratio of 2.9. The borders of the lesions are slightly indistinct. The largest lesion measures approximately 6.7 cm × 5.1 cm in cross-section. Multiple enlarged lymph nodes are observed in the hepatogastric ligament, showing increased radioactive distribution with an SUVmax of 16.9. The largest lymph node measures approximately 1.3 cm × 1.1 cm in cross-section (Figures 2A1–A8). According to the Barcelona Clinic Liver Cancer (BCLC) staging system, the patient is classified as stage B, which suggests treatment options such as transarterial chemoembolization (TACE), systemic therapy, or liver transplantation. However, since the patient’s A-fetoprotein (AFP) levels are not elevated, a diagnosis of hepatocellular carcinoma (HCC) cannot be confirmed. The possibility of a neuroendocrine tumor cannot be ruled out. So, on August 26, 2022, the patient underwent an [^18^F]AlF-OC PET/CT scan, which indicated a PHNET with abdominal lymph node metastasis. Multiple clustered and heterogeneous low-density lesions are observed in the left lobe of the liver, with SUVmax = 36.6, SUVmean = 16.3, and T/N = 4.0. The largest lesion measures approximately 8.3 cm × 6.6 cm in cross-section. Enlarged lymph nodes are visible in the hepatogastric ligament, showing increased radiotracer uptake with SUVmax = 37.9. The largest lymph node measures approximately 1.3 cm × 1.1 cm in cross-section (Figures 2B1–B8). The patient has been ultimately ruled out for hepatocellular carcinoma and metastatic liver neuroendocrine tumor, and the diagnosis is confirmed as PHNETs.

On August 30, 2022, the patient underwent a left hepatectomy, cholecystectomy, adhesiolysis for intestinal obstruction, partial gastrectomy, and intra-abdominal lymph node dissection under general anesthesia with endotracheal intubation. A firm mass was observed in the left liver lobe. Multiple enlarged lymph nodes were palpated along the lesser curvature of the stomach, showing a close relationship with the gastric wall, suggesting invasion into the gastric wall. Gastric wall resection and lymph node excision were performed. The surgery lasted approximately 310 min, with an estimated blood loss of about 200 mL. No blood transfusion was required.

**Figure 3:**
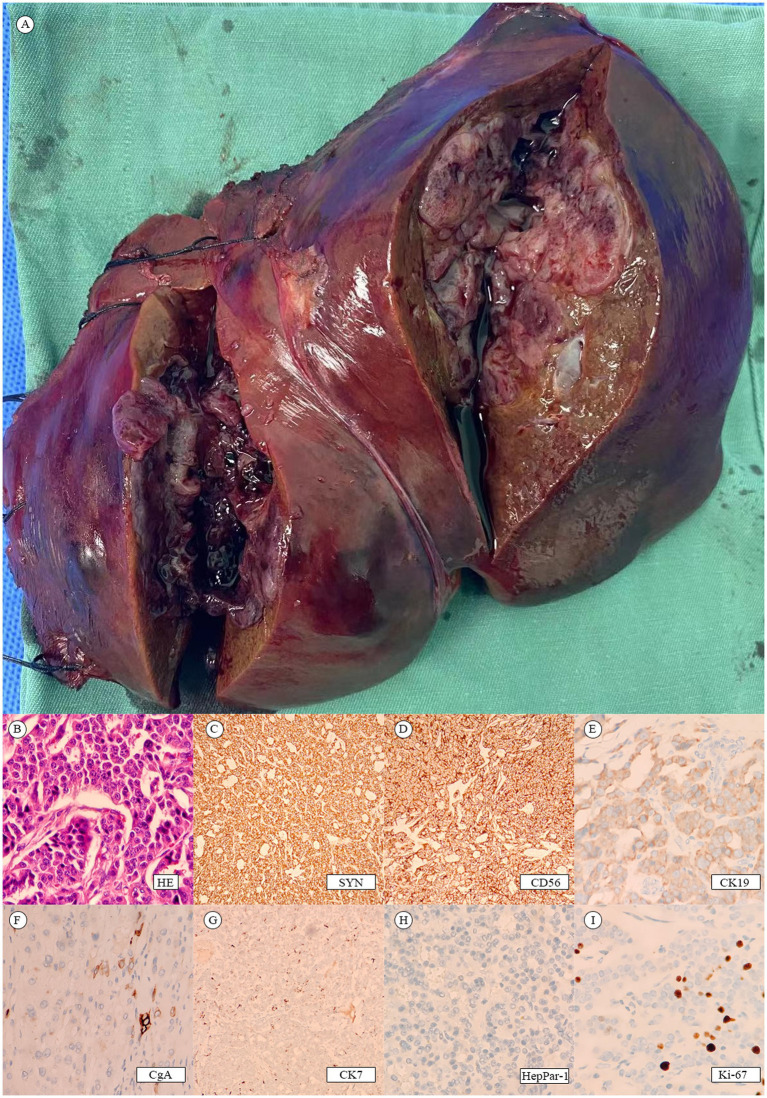
**(A)** Postoperative liver tumor. **(B–I)** Tumor pathology.

Postoperative pathology report: The left hemi-liver specimen reveals a malignant tumor (Figure 3A). Immunohistochemical analysis confirms it is a neuroendocrine tumor (NET, G3). The immunohistochemical markers show positive staining for Synaptophysin (Syn), CD56, CK19, and partially positive staining for Chromogranin A (CgA) and CK7. The markers HepPar-1, Glypican-3, CD10 are negative. Blood vessel staining with CD34 is positive. The Ki-67 proliferation index is 30% (Figures 3B–I).

## Discussion

NETs are a diverse group of tumors originating from neuroendocrine cells that can uptake and decarboxylate amine precursors. While commonly found in the stomach, intestines, and pancreas, the pancreas remains the most frequent site of occurrence. PHNETs, on the other hand, are relatively uncommon (1). PHNETs present a challenge regarding their non-specific clinical symptoms and limited diagnostic specificity through imaging examinations. Furthermore, the homogeneous nature of NETs poses challenges in differentiating between PHNETs and metastatic NETs in the liver.

Due to the limitations of conventional anatomical imaging techniques, the specific endocrine features of PHNETs cannot be easily visualized. As a result, distinguishing PHNETs from HCC can be challenging (6, 7). The overexpression of somatostatin receptors (SSTR) is a prominent feature of NETs. This provides a crucial target for diagnosing NETs and peptide receptor radionuclide therapy (PRRT), which utilizes peptide-receptor interactions for targeted treatment (8). PET imaging offers advantages such as high sensitivity and spatial resolution. Conventional [^18^F]FDG glucose metabolism PET/CT imaging plays a crucial role in clinically diagnosing and treating tumors. Furthermore, PET/CT targeted imaging using radiolabeled tumor-specific markers is vital for precise tumor diagnosis, staging, and personalized treatment planning. Commonly used radiolabels include [^18^F]F, [^68^Ga]Ga, and [^11^C]C.

In recent years, [^68^Ga]Ga-labeled somatostatin analogs (SSAs) for diagnosis and treatment have been increasingly widespread in clinical practice. Currently, commonly used in clinical practice are [^68^Ga]Ga SSA-PET, such as [^68^Ga]Ga-DOTATATE, [^68^Ga]Ga-DOTATOC, and [^68^Ga]Ga-DOTANOC, which have become the gold standard for the diagnosis of NETs (9, 10). However, [^68^Ga]Ga SSA-PET have their limitations, such as the higher positron energy of ^68^Ga, which can lead to inferior image quality compared to ^18^F. The advantages of [^18^F]AlF-OC include its shorter synthesis time, strong affinity to SSTR, good stability, and excellent imaging performance in neuroendocrine tumors (11). It significantly improves the tumor-to-background ratio, making the lesions more clearly visible.

In this article, despite the utilization of [^18^F]FDG PET/CT examination, the specific type of liver tumor remained indeterminate. However, the diagnosis was ultimately established using [^18^F]AlF-OC, enabling the development of a treatment plan primarily centered around surgical resection. This approach prevented misdiagnosis and ensured appropriate action (12).

Surgical resection remains the primary approach for treating primary lesions of NET G1 and G2. Preoperative evaluation should encompass various aspects, including tumor size, non-specific symptoms, endocrine function, the location of the primary lesion, and the extent of local invasion. Hepatic lobectomy is considered the most effective treatment for PHNETs, and in some cases, surgical intervention can lead to a complete cure with a favorable prognosis (13). Additionally, liver transplantation can be a viable option for select patients with PHNETs. Patients meeting certain criteria, such as well-differentiated NETs (Ki-67 < 10%), age < 55 years, absence of extrahepatic disease, complete resection of the primary tumor before transplantation, at least 6 months of disease stability before transplantation, and liver involvement <50%, can benefit from liver transplantation. Moreover, for unresectable PHNETs, TACE has shown promising therapeutic efficacy due to the rich vascularity and ischemia sensitivity of neuroendocrine tumors.

## Conclusion

In conclusion, PET/CT imaging, particularly with [^18^F]AlF-OC, plays a crucial role in diagnosing and managing PHNETs. The advantages of [^18^F]AlF-OC, including its shorter synthesis time, strong affinity to somatostatin receptors, and excellent imaging performance, contribute to improved visualization and accurate assessment of PHNETs. [^18^F]AlF-OC has significantly enhanced the tumor-to-background ratio, leading to a clearer identification of lesions. This imaging modality has proven valuable in guiding treatment decisions and avoiding misdiagnosis in challenging cases. Continued research and development in PET/CT imaging techniques, including using radiolabeled tracers, will further advance the field and improve patient outcomes in the management of PHNETs.

## Data availability statement

The original contributions presented in the study are included in the article/supplementary material, further inquiries can be directed to the corresponding author.

## Ethics statement

Written informed consent was obtained from the individual(s) for the publication of any potentially identifiable images or data included in this article.

## Author contributions

ZZ: Formal analysis, Visualization, Writing – original draft, Writing – review & editing. HL: Data curation, Formal analysis, Supervision, Writing – original draft, Writing – review & editing.
